# Wasting of the Roots of Teeth

**Published:** 1858-01

**Authors:** 


*Wasting of the Roots of Teeth.*—Dr. S. B. Hamlin, of Nashville, Tenn., presented to the senior editor, at the last meeting of the American Dental Convention, four upper incisors, which he had recently extracted from the mouth of a lady in consequence of their having become so much loosened as to be a source of constant irritation. The loosening of these teeth was occasioned by the destruction of the larger portion of their roots, caused, so far as we could learn, by disease of the alveolo-dental periosteum, but to what this was attributable, we were unable to ascertain. It may have been occasioned by the action of the lower teeth upon the upper. We have met with examples of the kind where it was clearly traceable to this cause.

